# Bioinspired microcone-array-based living biointerfaces: enhancing the anti-inflammatory effect and neuronal network formation

**DOI:** 10.1038/s41378-020-0172-0

**Published:** 2020-07-27

**Authors:** Hongxu Chen, Lulu Wang, Yi Lu, Xuemin Du

**Affiliations:** 1 Institute of Biomedical & Health Engineering, Shenzhen Institutes of Advanced Technology (SIAT), Chinese Academy of Sciences (CAS), Shenzhen, 518055 China; 2 The Brain Cognition and Brain Disease Institute, Shenzhen Institutes of Advanced Technology, Chinese Academy of Sciences; Shenzhen-Hong Kong Institute of Brain Science-Shenzhen Fundamental Research Institutions, Shenzhen, 518055 China

**Keywords:** Nanoscale devices, Nanoscale materials

## Abstract

Implantable neural interfaces and systems have attracted much attention due to their broad applications in treating diverse neuropsychiatric disorders. However, obtaining a long-term reliable implant-neural interface is extremely important but remains an urgent challenge due to the resulting acute inflammatory responses. Here, bioinspired microcone-array-based (MA) interfaces have been successfully designed, and their cytocompatibility with neurons and the inflammatory response have been explored. Compared with smooth control samples, MA structures cultured with neuronal cells result in much denser extending neurites, which behave similar to creepers, wrapping tightly around the microcones to form complex and interconnected neuronal networks. After further implantation in mouse brains for 6 weeks, the MA probes (MAPs) significantly reduced glial encapsulation and neuron loss around the implants, suggesting better neuron viability at the implant-neural interfaces than that of smooth probes. This bioinspired strategy for both enhanced glial resistance and neuron network formation via a specific structural design could be a platform technology that not only opens up avenues for next-generation artificial neural networks and brain-machine interfaces but also provides universal approaches to biomedical therapeutics.

## Neural networks: Effectiveness of a microcone array-based living interface

A microcone is a microscopic cone, and a system based on a microcone array (MA) has been successfully developed and found to be effective as a neural interface. Implantable neural interfaces have received great attention through their broad applications in treating various neuropsychiatric disorders. However, acute inflammatory responses have hampered efforts to design a long-term, reliable implantable interface. A team headed by Xuemin Du and Yi Lu at the Chinese Aecademy of Sciences, Shenzhen, China succeeded in producing an MA-based living interface, which significantly elevated the activity of neurons and alleviated the inflammatory response at the interfaces after 6 weeks’ implantation in mouse brains. The authors believe that the excellent neuron network formation of their system offers considerable potential as a platform technology for developing next-generation artificial neural networks and brain-machine interfaces and for use in biomedical therapeutics.

## Introduction

Implantable neural interfaces and systems have attracted much attention due to their broad applications in treating diverse neuropsychiatric diseases, including Parkinson’s disease, epilepsy, blindness, essential tremors, anxiety, and depression^1–8^. Conventional implantable devices for the central nervous system (CNS) are typically fabricated with polymers, organics, metals or their composites, which are very quickly identified as foreign materials after insertion, thus eliciting inflammatory responses^9–15^. These ongoing acute immunological processes cause glial encapsulation and neuronal loss around the implants, leading to a substantial reduction in the performances and even loss of functionalities of implantable devices (for example, increasing the electrode impedance and thus reducing/losing the signal-to-noise ratio during recording). Therefore, enhancing glial resistance to ensure long-term reliability is crucial for these implantable devices to reach their full potential^16,17^.

Fortunately, the development of implantable devices and surface modifications offers significant opportunities for improving implant-neural interfaces. For example, decreasing the dimensions and stiffness of implantable devices leads to a reduced mechanical mismatch between the implants and neural tissues, thus reducing tissue damage^1,10,13,15^. Additionally, modifications to the implant-neural interfaces can alleviate the inflammatory responses via surface immobilization (grafting or coating) with biocompatible macromolecules or polymers, controlled delivery of anti-inflammatory drugs or bioactive factors, and the incorporation of nanostructured materials^15,17–24^. These efforts have been conducted to improve the anti-inflammatory effects of implantable devices; however, these strategies still suffer from one or more of the following challenges in practical applications: (1) complex modification processes; (2) bioactive macromolecules and drug-loading/-releasing/-storage problems; and (3) inconsistent long-term anti-inflammatory effects. Therefore, it is highly crucial to develop a new strategy that can address the abovementioned issues.

Recently, the design of implanted devices has benefited from the merits and opportunities afforded by bioinspired design strategies^25–27^. These strategies include designing stimuli-responsive materials with dynamic stiffness for easy insertion of the devices, intelligent devices with shape-transformation abilities, and neuron-like probes^28–32^. In view of these successes in implementing bioinspired concepts in neural implants, it is noteworthy that bioinspired living biointerfaces that integrate rose-petal-like microcone structures and creeper-like neuron behaviors to promote anti-inflammatory effects have not been explored^1,9,12,15,33^. In this work, we develop a microcone-array-based living biointerface that matches the cellular feature sizes of neurons. We find that these rose-petal-like microcone structures not only ensure excellent cytocompatibility with neurons but also facilitate the formation of complex and interconnected neuronal networks. The specific size of the microcones ensures the preferential attachment of neurons (not astrocytes) and superior interactions among them. The rough surface morphologies of the microcones also help the outgrowth and arrangement of neurites. Furthermore, we verify the effectiveness of these biointerfaces after implanting microcone-array-based probes (MAPs) in the brains of mice for 6 weeks. We find significantly decreased glial encapsulation and neuron loss around the MAPs, indicating better neuron viability at the MAP-neural interfaces. To the best of our knowledge, this is the first living biointerface, which is fabricated merely with microcone-array-based structures, enhancing both anti-inflammatory effects and neuron network formation. Moreover, we believe that this strategy for fabricating bioinspired living biointerfaces can not only open up opportunities for next-generation artificial neural networks and brain-machine interfaces but also provide universal approaches to regenerative medicines.

## Results and discussion

**Fig. 1:**
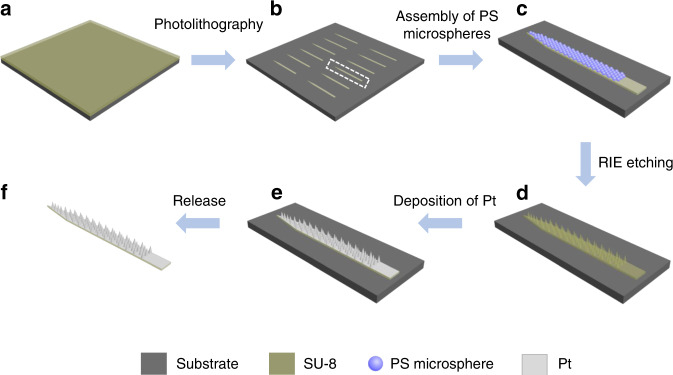
Schematic illustration of the fabrication process of Pt MAPs. **a** A layer of SU-8 photoresist was spin-coated onto a silica wafer. **b** Patterned SU-8 probes (15 mm in total length, 5 mm in tip length, 300 μm in maximum width, and 50 μm in thickness) were obtained after photolithography. **c** PS particles with a size of 5 μm were assembled on the surfaces of SU-8 probes. **d** SU-8 MAPs were fabricated via RIE etching. **e** A 200-nm-thick Pt layer was deposited onto the SU-8 MAPs by sputtering. **f** Pt MAPs were released from the silica wafer

**Fig. 2:**
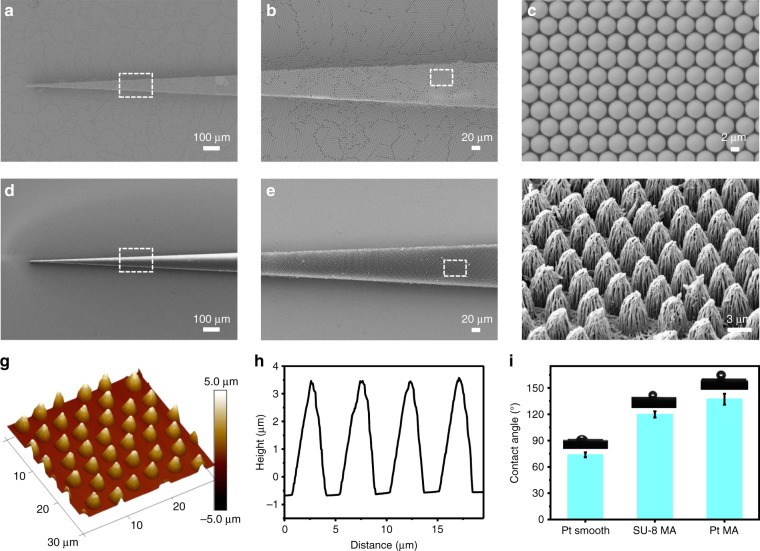
Physical characteristics of Pt MAPs. **a**–**c** Scanning electron microscopy (SEM) images of an SU-8 probe with a high-quality assembly of a two-dimensional (2D) hexagonal close-packed layer of PS particles (**b**); an enlarged view of the surface is also shown (**c**). **d**–**f** SEM images of a Pt MAP with periodically patterned microcones on the surface (**d**, **e**); an enlarged view of the surface is also shown (**f**). **g**–**i** An AFM image of the Pt MAP, the corresponding sizes (4.0 μm in height, 3.9 μm in maximum diameter, and 1.5 μm in spacing) (**h**) and the surface wettability (**i**)

In a typical experiment, probes with periodically patterned microcones were prepared by photolithography and colloidal lithography according to a modified procedure (Fig. 1)^34^. First, SU-8 probes with an optimized size (15 mm in total length, 5 mm in tip length, 300 μm in maximum width, and 50 μm in thickness) were fabricated by photolithography on the surface of a silica wafer (Fig. 1a, b, Fig. S1). These small probes facilitated the high-quality assembly of two-dimensional (2D) hexagonal-close-packed layers of polystyrene (PS) particles (with a size of 5 μm) at their surfaces via an interfacial method (Figs. 1c, 2a–c, Fig. S2)^34,35^. Subsequently, the SU-8 probes with PS assemblies were additionally treated with 7-min reactive ion etching (RIE), where the 2D PS colloidal layers were used as sacrificial masks to etch the SU-8 layer. After the PS colloidal masks were completely removed, SU-8 probes with periodically arranged microcone arrays (MAs) were obtained (Fig. 1d). Finally, these probes were released via a lift-off technique after a 200-nm-thick Pt layer was deposited by sputtering (Fig. 1e, f, Fig. S3).

The resulting probes were observed using field-emission scanning electron microscopy (SEM). As shown in Fig. 2d–f, the probe possesses rose-petal-like microcones, and the dimensions of the microcones are typically 4.0 μm in height, 3.9 μm in maximum diameter and 1.5 μm in spacing (Fig. 2f–h). It can be seen that the microcone surfaces are covered with hierarchical micro/nanogrooves, which is due to the synchronic etching process (Fig. 2f, Fig. S3). These rough structures not only ensure strong adhesion between the SU-8 and Pt layers but also result in different wettabilities. As shown in Fig. 2i, the static contact angles (CAs) of the water droplet on the Pt smooth film and SU-8 MA are 74° and 120° (CAs > 65°), respectively. It is known that increasing the surface roughness at the micro/nanoscales can result in a more hydrophobic surface for CAs > 65°^36–38^. Thus, the Pt MA becomes more hydrophobic (137°) after the deposition of the ~200 nm Pt layer onto the SU-8 MA surface, which further increases the roughness of the SU-8 MA. It is also found that the obtained Pt MAPs are still flexible even when deposited with a Pt layer, which can guarantee their biocompatibility and conductivity (Fig. S4). Compared with previously demonstrated implantable probes^17^, these flexible Pt MAPs with hierarchical micro- and nanostructures, which may drastically increase the contact area and friction between microcones and tissues, will result in a more stable interface (Fig. 2e–i, Fig. S3, Fig. S4).

**Fig. 3:**
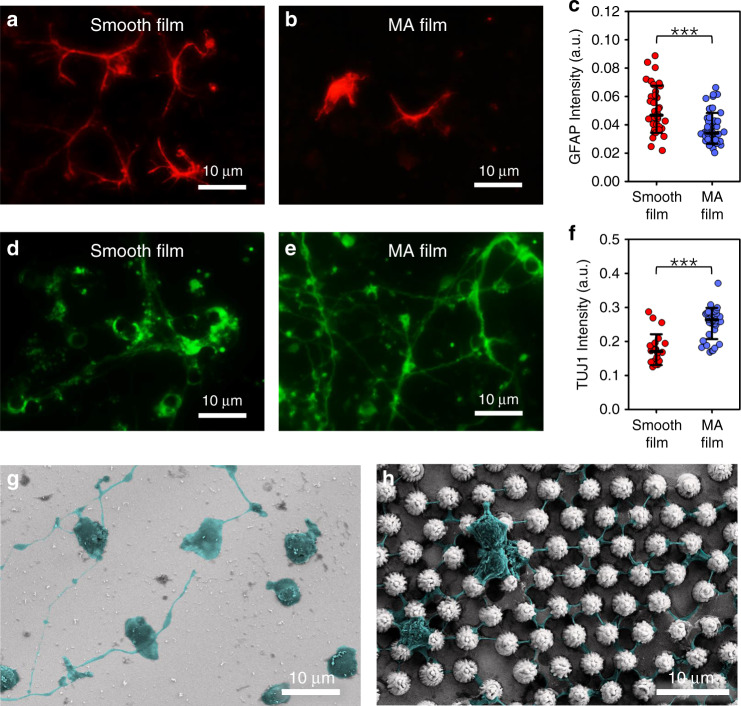
Hippocampal cells cultured on Pt smooth film and Pt MA film on day 10. **a**–**f** GFAP (**a**, **b**) and TUJ1 (**d**, **e**) immunostaining of Pt smooth film (**a**, **d**) and Pt MA film (**b**, **e**). GFAP (**c**, Pt smooth film, *n* = 36 images from six samples; Pt MA film, *n* = 51 images from nine samples) and TUJ1 immunoreactivity (**f**, Pt smooth film, *n* = 22 images from five samples; Pt MA film, *n* = 35 images from seven samples) were compared using two sample *t*-tests, shown as the means ± SEM (***p < 0.005). **g**–**h** SEM images of hippocampal neurons cultured on Pt smooth film (**g**) and Pt MA film (**h**); neurons and neurites are shown in pseudocolors

To quantitatively test the cytocompatibility of the microcone-array-based surfaces, mouse hippocampal primary neurons were cultured on the surfaces of Pt smooth films and Pt MA films for 10 days (Fig. 3). Astrocytes were stained with glial fibrillary acidic protein (GFAP, red), while neurons were stained using neuronal class III β-tubulin (TUJ1, green). On the surfaces of the Pt smooth films (Fig. 3a), the astrocytes exhibited large body sizes and cell domains, indicating a moderate gliosis. However, astrocytes on the Pt MA films showed notably reduced cell body sizes and fibrous morphologies with notably reduced cell volumes (Fig. 3b). The GFAP intensity on the Pt MA films was significantly lower (*P* < 0.005) than those on the Pt smooth films (Fig. 3c), suggesting a glial-resistant characteristic of the Pt MA films. Neurons on the Pt smooth films showed neurite outgrowth and the formation of neural connections (Fig. 3d). Notably, on the surfaces of the Pt MA films, the neurons exhibited much denser and highly ordered extending neurites, which formed complex and interconnected neuronal networks (Fig. 3e). The expression of fluorescent TUJ1 on the Pt MA films was significantly higher (*P* < 0.005) than that on the Pt smooth films (Fig. 3f), implying that the MA structure facilitates the attachment and differentiation of neurons. Because the formation of highly complex and interactive neuronal networks is essential for all brain functions, the Pt MA structure exhibits great potential and advantages in applications involving the dissection and modulation of neural circuits^39^.

Furthermore, SEM images were obtained to investigate the influence of microcones with hierarchical micro/nanogrooves on neuronal cells (Fig. 3g, h). Neurite extensions were observed on the surfaces of both Pt smooth (Fig. S5) and Pt MA films. However, it is obvious that the morphology of neurons is better on the Pt MA surfaces, and the neurites, which behaved as creepers wrapped tightly around the periodically arranged microcones, are notably thicker and longer than those on the Pt surfaces. This observation may be attributed to the rough micro/nanostructures at the microcone surfaces, which facilitate the adhesion and twining of neurites. Consequently, these creeper-like neurites were tightly attached to and wrapped around the microcones, forming a uniformly structured neuronal network. Collectively, the results suggest that the three-dimensional micro/nano morphology of the Pt MA surface may contribute to the excellent cytocompatibility with neurons (not astrocytes) and the formation of highly interconnected neuronal networks, ensuring a potential glial-resistant effect for in vivo applications.

Normally, an inflammatory response is quickly elicited after a neural probe is implanted into the CNS, which leads to the formation of astroglial encapsulation. This encapsulation separates the probe from the targeted neurons and hinders signal transformation at the implant-neural interface. Worse still, the inflammatory response may also lead to a loss of neurons at the implantation site, which further deteriorate the performances of the probes^40^. An in vitro study previously revealed the good cytocompatibility and glial-resistant characteristics of Pt MA films; hence, to evaluate the chronic performances of the MAs, brain slides from mice receiving Pt smooth probes and Pt MAPs were further studied with immunochemistry after a 6-week implantation (Fig. S6).

**Fig. 4:**
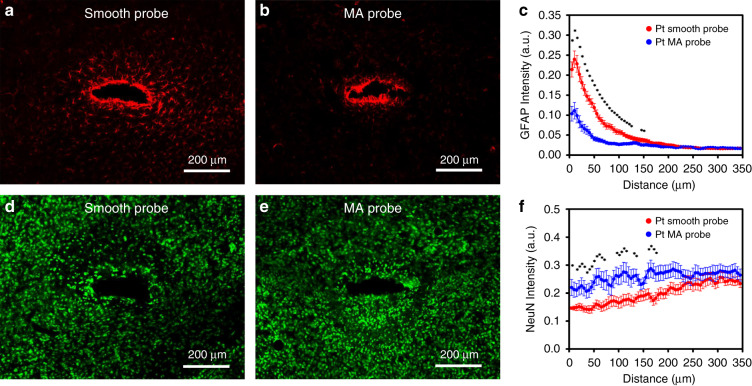
Inflammatory response and neuronal survival around implants at 6 weeks after implantation in a mouse brain. GFAP (**a**, **b**) and NeuN (**d**, **e**) immunostaining of Pt smooth probes (**a** and **d**, *n* = 18) and Pt MAPs (**b** and **e**, *n* = 16). Quantitative comparisons of GFAP (**c**) and NeuN (**f**) immunoreactivity between Pt smooth probes and Pt MAPs were performed by using intensity profiles as a function of the distance from the implant interface, shown as the means ± SEM (*p < 0.05, independent-samples *t*-test)

The inflammatory response at the implant-neural interface was characterized by the expression of GFAP. It was found that the reactivated astrocytes accumulated and occupied the zone around the implantation site of the Pt smooth probe (Fig. 4a). A much weaker expression of GFAP was observed around the implantation site of the Pt MAP (Fig. 4b). Quantitative analyses of the GFAP immunohistological intensity profiles of the Pt smooth probe and Pt MAP as a function of the distance from the interface are shown in Fig. 4c. The average thickness of the astroglial encapsulation in the Pt smooth probe group was ~100 μm, while in the Pt MAP group, it was only ~50 μm. The average intensity of GFAP immunostaining in the Pt MAP group was significantly lower (*p* < 0.05) than that of the Pt smooth probe group along the length of ~120 µm relative to the implant interface. The result suggests a significantly decreased GFAP-positive zone at the implant interface, which is in accordance with the results in cell culture studies. The neuronal densities in the vicinity of the implant were studied by neuronal nuclei (NeuN) immunostaining. Neuronal loss around the implantation site was especially severe in the Pt smooth probe group (Fig. 4d). However, no notable loss of neurons was found in the Pt MAP group (Fig. 4e). Quantitative analysis of NeuN intensity (Fig. 4f) revealed a severe loss of neurons within an average distance of ~130 µm from the implantation site in the control group. However, in the Pt MAP group, the average distance decreased to ~40 µm. The statistical results show that the average intensity of NeuN immunostaining in the Pt MAP group was remarkably higher than that of the Pt smooth probe group (*p* < 0.05 within ~130 µm), suggesting better neuron viability at the Pt MAP interfaces. These findings are consistent with the results of in vitro studies, which suggests that the Pt MAP can alleviate the host-interface response and enables neurons with implantable devices to be used in chronic applications in vivo^41^.

## Conclusions

In summary, MA-based living biointerfaces represent an unprecedented attempt at bioinspiration as tools for the design of implantable devices for neurotechnology. Compared with other state-of-the-art neural interfaces, MAs with hierarchical micro/nanostructures match the cellular feature sizes of neurons, thus ensuring much denser extending neurites and forming highly interconnected neuronal networks, which behave similar to creepers. Interestingly, the featured sizes and roughness of the MA facilitate the attachment and differentiation of neurites yet resist the adhesion of astrocytes, suggesting the significant enhancement of glial-resistant effects compared with previous works. Notably, the MAP is a substrate for the attachment of neurons, and the pattern design of MAs could help form patterned neuron networks. This suggests the potential of the MAP in the reconstruction and study of neuronal networks and the precise dissection and modulation of neural functions. Via the combination of other functional materials, our bioinspired living biointerface strategy for both enhanced glial-resistant effects and neuron network formation could be a platform technology that not only opens up avenues for next-generation artificial neural networks and brain-machine interfaces but also provides universal approaches to biomedical therapeutics^1,27,42–47^.

## Materials and methods

### Fabrication of MA structures

SU-8 photoresist (SU8-3050, NIPPON KAYAKU Co. Ltd.) was spin-coated on a silica wafer to a thickness of ~50 μm, prebaked sequentially at 95 °C for 10 min, and then patterned by photolithography (EVG 610, Austria). Afterwards, the SU-8 photoresist was postbaked at 95 °C for 5 min, developed for 2 min (SU-8 Developer, IPPON KAYAKU Co. Ltd.), rinsed with isopropanol, and dried with N_2_. Then, the SU-8 films or probes were treated with oxygen plasma (Gatan Solarus Model 950) for 5 min. Subsequently, a hexagonally close-packed PS microsphere layer (5 μm) was assembled on the as-prepared SU-8 films or probe surfaces by the interface method^34,35^. After drying in air at room temperature, the MAs were fabricated via ICP-RIE (ICP-RIE DSE200S system). The ICP-RIE process was performed at a pressure of 20 mTorr, a flow rate of 100 sccm (oxygen), an Src RF power of 300 W, and a Bis RF power of 100 W. The sputter deposition of the thin Pt layer was carried out using a magnetron sputtering system (TRP-450, Shenyang Scientific Equipment Corporation, China) in an argon atmosphere at a low pressure (5 × 10^−4^ Torr). A 200-nm Pt layer was obtained in 500 s. Then, Pt MA films or probes were released after being immersed in 2 wt% sodium hydroxide solution for 6 h to etch the silicon layer. After washing with ultrapure water, the Pt MA films or probes were dried in a stream of nitrogen gas.

### Characterizations

The morphologies of the as-prepared SU-8 films and probes with MAs were recorded by a digital camera (Canon, 7D Mark II) and an optical microscope (Nikon Ni-U). The morphologies of the MAs and cells were determined by field-emission scanning electron microscopy (FE-SEM, ZEISS SIGMA 300). The detailed morphology and spacing of the microcones were characterized by atomic force microscopy (AFM, Bruker Dimension Icon).

### Wettability assessment

Static CAs were measured using a CA meter (DSA25, Kruss, Germany) at room temperature, and each CA value was measured at least five times. The stabilized shapes of the water droplet on the samples were recorded by a digital camera.

### Preparation of the samples

Pt smooth and MA films (10 mm × 10 mm) and probes (15 mm in total length, 5 mm in tip length, 300 μm in maximum width, and 50 μm in thickness) were used for the cell culture and implantation studies, respectively. All samples were obtained prior to use. All samples for the cell culture and implantation studies were cleaned in ultrapure water three times and sterilized using ultraviolet (UV) radiation prior to use.

### Cell cultures

All samples were coated with 10 mg/ml of poly-L-lysine for 6 h prior to primary hippocampal neuron cultures. Cells from the hippocampus of newborn wild-type C57 mice were seeded at a density of 5 × 10^4^ cells/ml in neurobasal medium containing 2% B27, 2 mM L-glutamine, 100 U/ml penicillin, and 100 mg/ml streptomycin. The cultured cells were kept at 37 °C in a humid atmosphere with 5% CO_2_, with half of the cell culture medium replaced every 4 days. At day 10, GFAP (abcam, USA) combined with beta III-tubulin (Tuj1, abcam, USA) was used to identify the glial cells and neurons, respectively. The cultured cells were incubated in 0.1 M PBS for 10 min and fixed with 4% PFA for 20 min. Then, the fixed cells were treated using 0.1% PBST (PBS containing 0.1% Triton-100) for 5 min and incubated with PBS containing 0.1% BSA for 40 min, followed by incubation with primary and secondary antibodies. After that, the cells were washed with 0.1 M PBS, and the fluorescence images were observed and taken using an inverted fluorescence microscope (Zeiss Axio Imager A2). A field-emission scanning electron microscope (SEM, Carl Zeiss, Germany) was used to investigate the neurite outgrowth and neural network formation of cultured cells. Samples were dehydrated by a freeze dryer for three days and coated with thin layers of gold prior to SEM observation.

### Implantation study

All procedures were conducted using sterile techniques in accordance with protocols approved by the Ethics Committee for Animal Research, Shenzhen Institutes of Advanced Technology, Chinese Academy of Sciences. Adult male C57 mice weighing 20–24 g were used for the chronic implantation experiment. The mice were housed under a 12-h light/dark cycle and provided with food and water ad libitum. After being anaesthetized with 1% phenobarbital sodium solution (1 ml/100 g), the animals were immobilized in a stereotaxic frame for sample implantation. A mid-sagittal incision was made at the scalp, and two holes were carefully created at locations −2.5 mm anterior and ±1.5 mm lateral to the bregma in each animal. Two groups of implants (including Pt smooth probes and Pt MAPs) were then slowly implanted into the brains. Afterwards, each implant was fixed to the skull with dental cement, and the skin was sutured shut with monofilament nylon.

The mice were sacrificed for immunohistological analysis 6 weeks after implantation following previous works. Briefly, mice were perfused transcardially with 0.1 M PBS followed by 4% paraformaldehyde in 0.1 M PBS, and the brains were removed and fixed at 4 °C for 2 days. Subsequently, the block tissue around the implant was paraffin embedded, and horizontal sections (35-μm thick) were taken from all brains. GFAP (abcam, USA) and NeuN (Millipore, USA) were used to label astrocytes and mature neurons, respectively. Fluorescent images were obtained using an inverted fluorescence microscope (Olympus VS120). Quantitative analysis was performed using custom software developed in MATLAB (MathWorks, USA). The staining intensity of GFAP and NeuN was calculated as a function of the distance up to the implant surface. The average intensity profiles of the analyzed area within a distance of 350 µm from the implant-neural tissue interface are shown. All data were presented as the mean ± standard error of the mean (mean ± SEM). The staining intensity of different implants at the same distance was analyzed using independent-sample *t*-tests with SPSS 16.0 (SPSS, USA). The *t*-test significance between groups was also presented as a function of the distance up to the implant site.

## Supplementary information

Supplementary Information1046
