# Supplementary material for: Bioinspired microcone-array-based living biointerfaces: enhancing the anti-inflammatory effect and neuronal network formation
Source: Microsyst Nanoeng. 2020 Jul 27;6:58. doi: 10.1038/s41378-020-0172-0 (PMC8433467; doi:10.1038/s41378-020-0172-0)
Supplement: Supplementary file 1 — Supplementary Information1046 [file 41378_2020_172_MOESM1_ESM.docx]

**Supplementary Information**

**Bioinspired microcone-array-based living biointerfaces: enhancing the anti-inflammatory effect and neuronal network formation**

Hongxu Chen^1^, Lulu Wang^2,3^, Yi Lu^2^, Xuemin Du^1^

^1^Institute of Biomedical & Health Engineering, Shenzhen Institutes of Advanced Technology (SIAT), Chinese Academy of Sciences (CAS), Shenzhen 518055, China ^2^Brain Cognition & Brain Disease Institute, Shenzhen Institutes of Advanced Technology (SIAT), Chinese Academy of Sciences (CAS), Shenzhen 518035, China ^3^Shenzhen College of Advanced Technology, University of Chinese Academy of Sciences, Shenzhen 518055, China

These authors contributed equally: Hongxu Chen, Lulu Wang


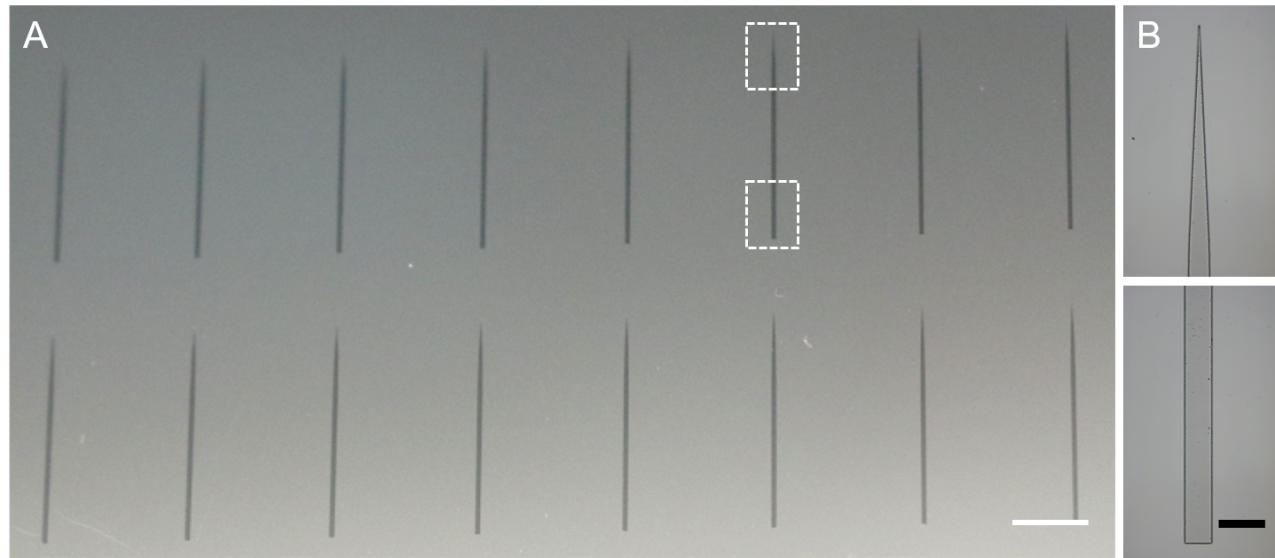


**Fig. S1** (A) Images of the SU-8 probe array and enlarged views of the morphologies (B). The scale bars are 500 μm.


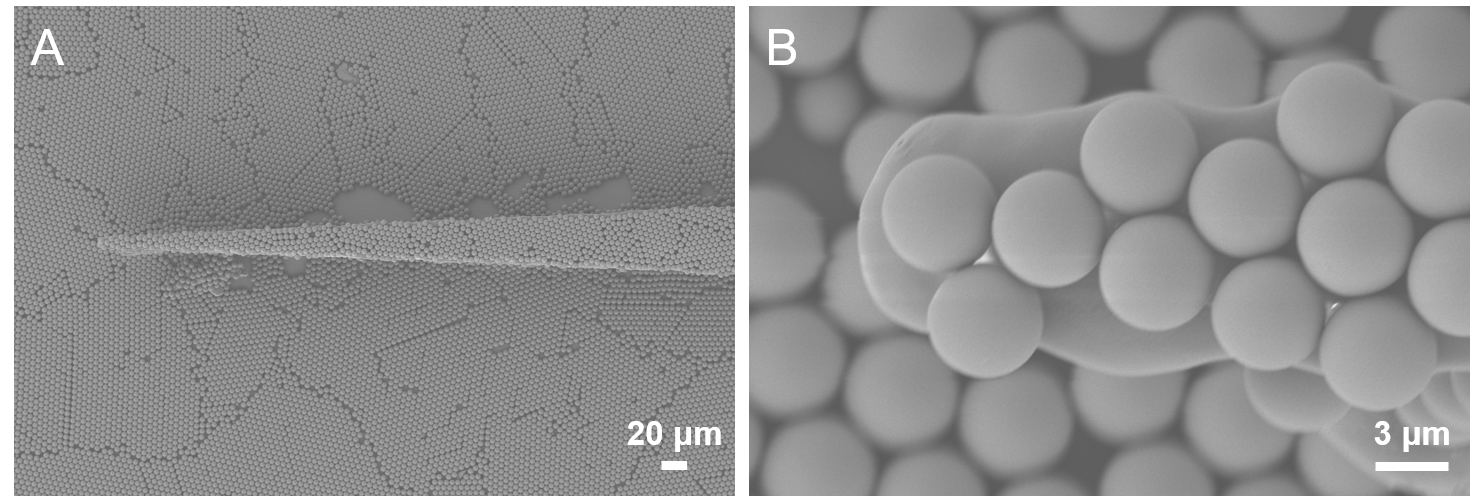


**Fig. S2** (A) SEM images of an SU-8 probe with hexagonally close-packed PS microspheres on the surface, and an enlarged view of the surface (B).


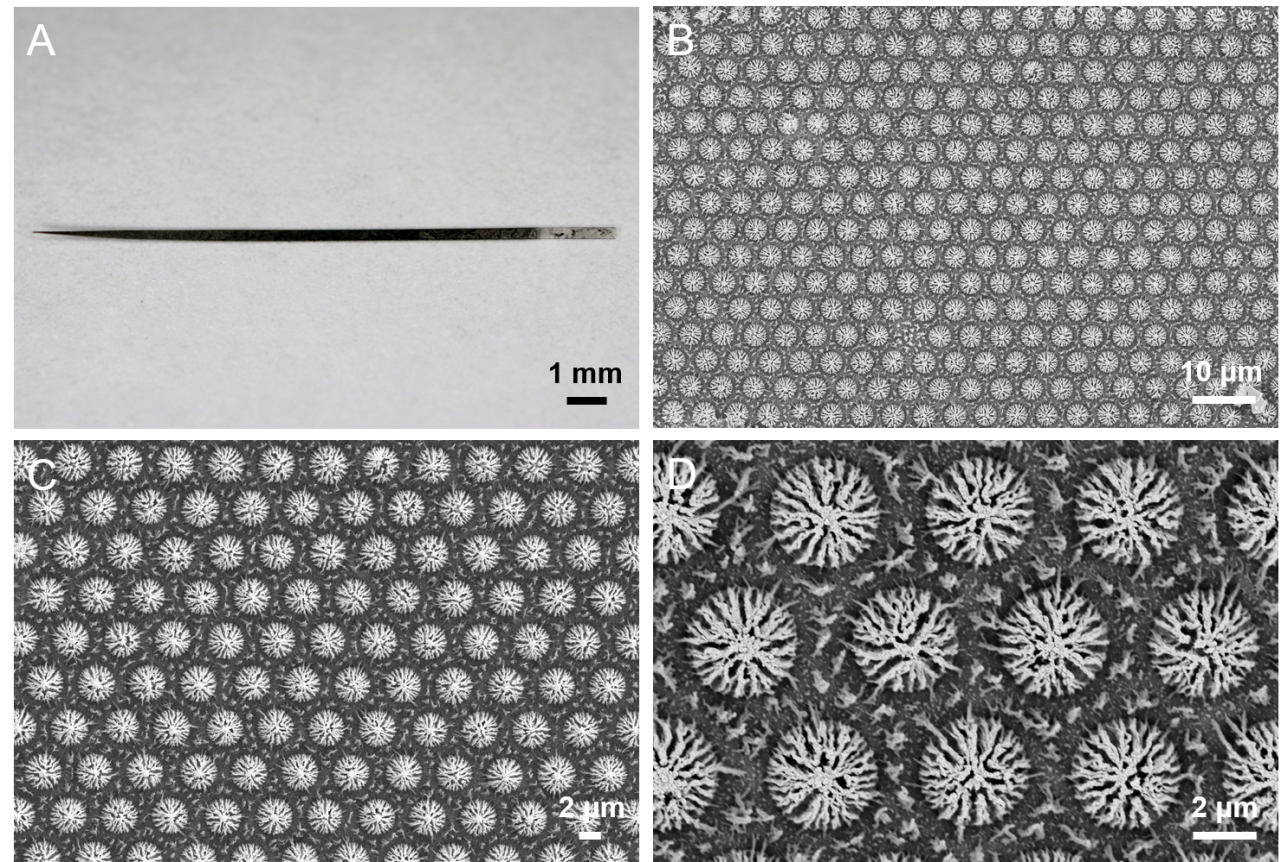


**Fig. S3** (A) An image of a Pt MAP, and enlarged SEM images of the surface (top-view morphologies) (B-D).


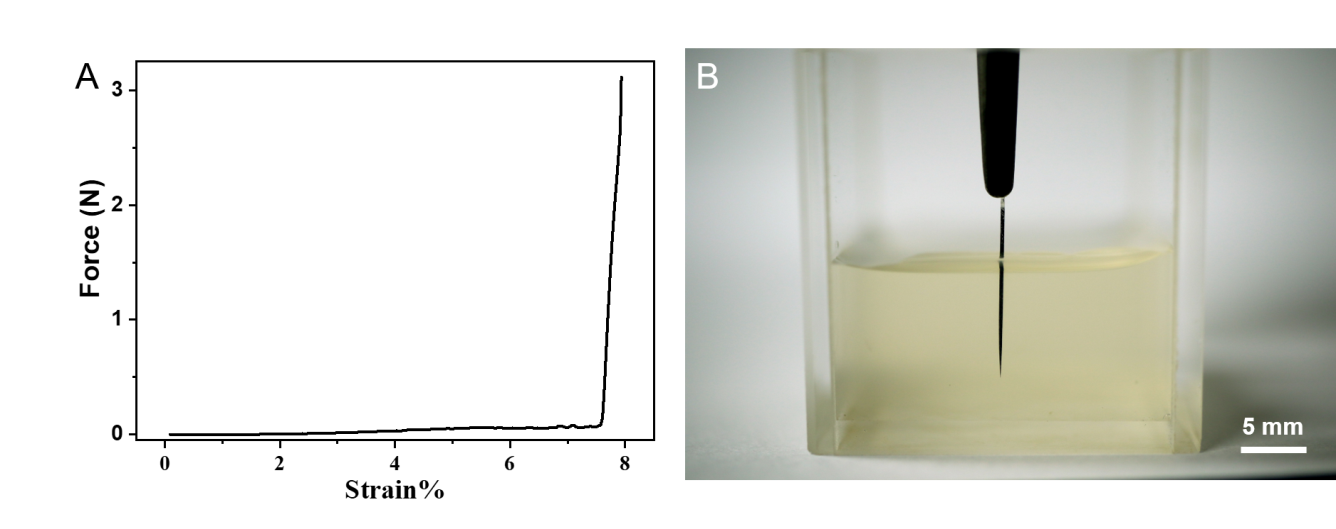


**Fig. S4** (A) Force-strain curve of a Pt MAP. The bending stiffness of the Pt MAP is 100 MPa. (B) Image of a Pt MAP inserted into an uncured sodium alginate (5.0 wt%) precursor.


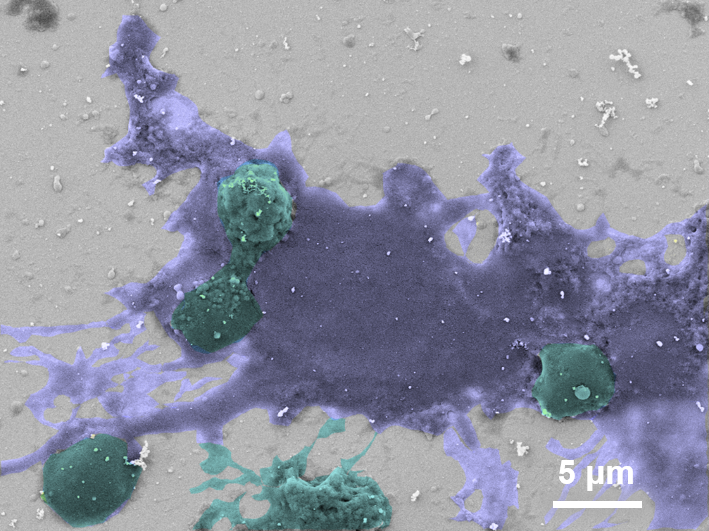


**Fig. S5** A representative SEM image of hippocampal cells cultured on a Pt smooth film. Neurons (blue) and astrocytes (purple) are shown in pseudocolors.


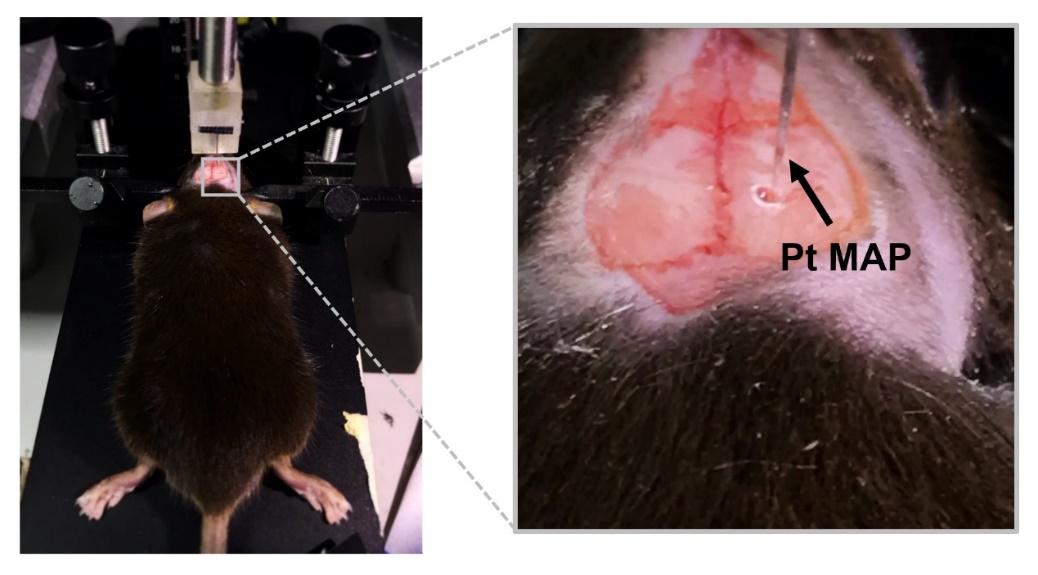


**Fig. S6** Representative images of a Pt MAP inserted into the hippocampus of a C57 wild-type mouse.
